# The Brain Effective Connectivity of Chinese during Rhyming Task

**DOI:** 10.1371/journal.pone.0162158

**Published:** 2016-09-01

**Authors:** Linlin Zhu, Zhendong Niu, Yaoxin Nie, Yang Yang, Ke Li, Zhen Jin, Jieyao Wei

**Affiliations:** 1 School of Computer Science and Technology, Beijing Institute of Technology, Beijing, China; 2 Department of Linguistics, University of Hong Kong, Hong Kong; 3 The 306th Hospital of PLA, Beijing, China; Xuanwu Hospital, Capital Medical Universty, CHINA

## Abstract

With regard to brain language processing, the activation patterns have been well studied, and recently there are great interest in the connectivity models. The crucial brain areas for phonological processing involves left inferior frontal gyrus (LIFG), left inferior parietal lobule (LIPL) and left posterior middle temporal gyrus (LpMTG). Specially in Chinese processing, the left middle frontal gyrus (LMFG) is considered as an essential region. However, the connectivity pattern among these brain areas is not well understood. In this study, a rhyming experiment of Chinese was conducted, and the Dynamic causal modeling (DCM) and the Bayesian model selection (BMS) were used to examine the interaction between brain regions and choose the best model for rhyming task of Chinese. By examining the interactions, it was found that LMFG exerted inhibitory modulation on LIPL and LIFG; the phonological processing enhanced the connection from LIPL to LIFG and LMFG, which suggested the important roles of these connections for the increased phonological load; And LpMTG modulated LIFG and LMFG negatively, and LIPL positively under rhyming judgment task.

## Introduction

Different language processing models have been put forth to describe the time-course of reading in alphabetic language [1, 2]. The most popular language processing model is the dual-route model [3–6]. But, Chinese, as a logographic language with single syllable per character, is different from alphabetic language such as English in various aspects. The brain mechanism of Chinese understanding differs from alphabetic language [7, 8]. Tan et al. [8]conducted a meta-analysis to examine phonological processing of written word forms in Chinese and English and found both Chinese and English phonological processing involved in activation in left parietal lobule (LIPL), left inferior frontal gyrus (LIFG) and bilateral occipitotemporal regions; and the left middle frontal gyrus (LMFG) was proposed to play an crucial role in the Chinese processing. Chen et al. [9, 10] established two priming-category judgment experiments and found that the phonological code and semantic code were active in a parallel mode. Thus, when reading printed Chinese, the functional connectivity in the brain may differ from reading written English. In this study, we used dynamic causal modeling (DCM) [11]) and Bayesian model selection (BMS) [12, 13]to find the best effective connectivity model during rhyming tasks of Chinese in visual modality.

In phonological processing studies, LIPL, LIFG and LpMTG are important areas. Moreover, LMFG is important for the Chinese processing. Despite growing number of studies in this field, the modulatory effect of the phonological task on the connections between these regions is still unclear.

LMFG (BA 9) is an important area for processing written Chinese. It was activated in homophone judgment [14–17] and rhyming decision tasks [18–20] except for part of Cao’s work [21]. In Cao et al., (2011), LMFG was activated in spelling task in the contrast adult > children. These studies indicated that LMFG involved in homophone, rhyming and orthography processing. LMFG was also activated in covert naming task [22–24], read aloud task [17]as well as lexical decision [25]. When performing vowel and tone production, LMFG was also activated [26]. Obviously, LMFG plays a key role in the phonological processing of Chinese, but the connectivity between this area and others is still unclear.

LIFG was activated in all Chinese phonological studies [14–16, 18–27]. In clinical studies, LIFG was also important. Damage to LIFG (Broca’s area) can cause non-fluent aphasia (Broca’s aphasia). The Broca’s aphasia patients suffer from agrammatic, halting speech. And, intonation and stress patterns are deficient. The researches about LIFG aphasia mainly focus on semantic processing. Many researchers propose that LIFG is for verb and grammar processing [28, 29]. Hickok denoted that motor speech system including Broca’s area may play a role in orthographic decoding or in auditory-visual matching of phonological forms [30]. In recent years, a research noted that cerebral circuits underlying noun and verb processing are not spatially segregated [31]. Vigliocco’s[32] critical review suggested that it was a confusion to separate the concept of verb and nouns, grammatical class should distinguished from semantic. In contrast to the semantic processing pattern, rhyming task activated the dorsal part of LIFG [18, 33]. And the dorsal portion of the LIFG may be involved in phonological processing [34–36]. Liu et al. [18] proposed that the dorsal part of LIFG was involved in strategic phonological processing as greater activation in this area was correlated with word pairs that had conflicting segmental and tonal information.

LIPL and adjacent supramarginal gyrus were activated in a lot of Chinese phonological studies. The activation varied task by task. The paradigms can be classified as follows: covert naming task [23, 24], read aloud task [27], tone and vowel production task [26], rhyming judgment task [19, 21], lexical decision [25] and homophone judgment [16]. Studies of Chinese generally support the role of the LIPL in phonological processing, but the interpretations are various. Some involved in the conversion between orthography and phonology [19] or visuospatial analysis when mapping from orthography to phonology [33]and others have argued that it is involved in short-term maintenance of phonological codes [8, 18]. Even though the role of IPL is controversial for phonological processing, LIPL seems to be due to the primary processing of phonology. Accordingly, LIPL is assigned as the driven input of the network in this study.

LpMTG and left posterior superior temporal gyrus (LpSTG) were activated in many studies [17, 22–25]. In these studies, some lexical parameters of Chinese such as character frequency were controlled strictly. These studies mentioned above did not adopt any phonological decisions as their experimental paradigms. Instead, they adopted a word naming or converted naming paradigms which minimized the nuisance factors that affected the language processing. In many language studies, LpMTG was always regarded as an important area for lexical-semantic processing [19, 37–40]. In a DTI research of the human brain’s language pathways, superior temporal gyrus (STG) terminations were strongly left lateralized and overlapped with phonological activations, but LpMTG terminations were overlapping with left lateralized lexical-semantic activations. Moreover, a research on processing of sub-syllabic speech units in the posterior temporal lobe showed that the MTG activation represented phonetic/phonological processing [41]. In addition, LpSTG was involved in phonological processing [2, 4, 6, 42–44] as well as LpMTG was involved in orthography-phonology mapping [45–47]. In this study, we expect activation in LpMTG, when adjusting some lexical parameters in the rhyming task, and explore the connections between LpMTG and other areas for rhyming tasks.

In recent years, there are increasing studies using DCM [11, 48] to explore the effective connectivity for language processing, with phonological tasks [49–51] and different tasks (i.e. spelling versus rhyming judgment, phonological versus lexical judgment) [49, 52–55]. Bitan et al. used DCM to estimate the effective connectivity with rhyming judgment and examine the development characteristics. Results showed that the phonological task modulated the connections from fusiform gyrus (FG), intraparietal sulcus (IPS), IFG to lateral temporal cortex (LTC) and from FG, LTC, IPS to IFG[49]. With the development contrast, the LTC played an increasingly important role [50]and the modulatory effect from IFG to IPS increased [51]for phonological task. In a recent study with the lexical decision task of Kanji and Hiragana, the effective connectivity was examined. The authors found reading Hiragana increased connection from IFG to supramarginal gyrus (SMG) which may be due to the top-down modulation to guide processes of phonological assembly or phonological working memory [56]. Though more and more studies focus on the effective connectivity of language processing, the connectivity pattern of Chinese phonological processing remains largely unknown.

In this study, we used DCM and BMS to examine the interaction between brain regions and favor the best model for rhyming task of Chinese. Particularly, we focused on the interaction between four regions, which were LMFG, LIPL, LIFG and LpMTG. By examining the interactions, we would demonstrate the roles of each area. Especially, by examining the connections between LMFG and other areas, we would give a probable explanation of the function of LMFG in Chinese phonological processing.

## Materials and Method

### Participants

Nineteen adults, 9 males and 10 females, 23–28 years of age, (mean age, 25±1.45 years), participated in this study. All the participants met the following criteria: 1) native Mandarin Chinese speakers, 2) right-handed, 3) free of neurological disease or psychiatric disorder, 4) no attention deficit hyperactivity disorder, 5) no learning disability, 6) All had normal or corrected-to-normal vision. After receiving the detailed information of experimental procedure and administration, all participants granted written informed consent. This experiment was approved by the Academic Program Committee of the laboratory of computer software in Beijing Institute of Technology.

### Stimuli and tasks

**Fig 1:**
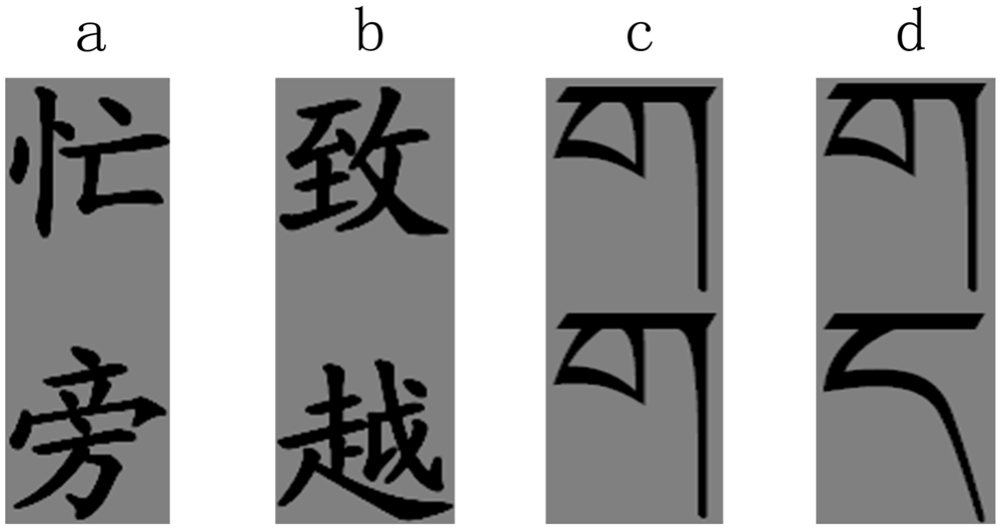
Examples of experimental stimuli. (a) Rhymed pairs which are pronounced /mang2/ (up) and /pang2/ (down). (b) Unrhymed pairs which are pronounced /zhi4/ (up) and /yue4/ (down). (c) Orthography matched Tibetan symbols. (d) Orthography unmatched Tibetan symbols.

Two types of stimuli were applied in this study. In the rhyming task, subjects were required to judge whether the two presented Chinese characters rhymed. In the perceptual control task, two Tibetan symbols were presented. Participants were required to determine whether the symbols have the same orthography form. The Tibetan symbols and Chinese characters were matched on their positions and sizes to eliminate nuisance effects. Fig 1 illustrates the examples of experimental items.

Previous studies have examined variables which affect the word/character naming latency in Chinese and have shown that the lexical variables such as frequency, phonetic regularity, the number of strokes and the number of components, are important for Chinese word naming. To minimize the correlations among these variables, we selected Chinese characters from the Chinese Single-character Word Database (CSWD) [57] and the online corpus (www.cncorpus.org). The first database is comprised of 16 variables for about 2390 Chinese characters which contains almost all the single-character nouns, verbs, and adjectives in modern Chinese. These characters were selected from the Language Corpus System of Modern Chinese Studies (LCSMCS) [58]. The second database offers the high frequency (more than 50 per 20 million) characters. Based on the previous behavior study [57], the characters in the study were balanced against word frequency, cumulative frequency, number of strokes and visual complexity.

### Experimental procedure

During the fMRI scanning, a block design was adopted. There were eight task blocks in the run, four blocks for the rhyming judgment and another four blocks for the Tibetan orthography judgment. During each block, an instruction of the block was briefly presented for 3 seconds followed by 10 trials. Each trial lasted 3 seconds, in which the stimulus was presented for 1 second followed by 2 seconds blank screen. Participants were asked to make their judgment as soon as the screen turned to blank. The 10 trials in one block were balanced in 5 true matching and 5 false matching. The true matching and false matching pairs in a block were mixed in a random order, and the order of the blocks was pseudo random. Before each task block, a fixation (“+”) was presented for 15 seconds. The subjects were asked to stare at the “+” in the middle of the screen. At the beginning of the run, the fixation was presented for 18 seconds. The run lasted for 387 s.

Participants viewed visual stimuli that were projected onto a screen via a mirror attached to the inside of the head coil, and were asked to give a response by pressing buttons below their thumbs, with right thumb represented “yes” and left thumb represented “no”.

### Image acquisition

All images were acquired using a Siemens Tim Trio 3T scanner with echo planar imaging (EPI) method. The following scan parameters were used: time repetition (TR) = 3000 ms, time echo (TE) = 30 ms, flip angle = 90°, matrix size = 64×64, field of view = 210×210 mm^2^, slice thickness = 4 mm, number of slices = 36. These scanning parameters resulted in a 3.28×3.28×4 mm^3^ voxel size. A high-resolution, T1-weighted three dimensional image was also acquired (TR = 2300 ms, TE = 2.98 ms, flip angle = 9°, matrix size = 256×256, field of view = 240×256 mm^2^, slice thickness = 1 mm, number of slices = 176). These parameters resulted in a 1×1×1 mm^3^ voxel size.

### fMRI Data Analysis

Data preprocessing and statistical analyses were performed using SPM8 (Statistical Parametric Mapping) (http://www.fil.ion.ucl.ac.uk/spm/). The first four time-points of the functional images were discarded. Then the images were realigned to the fifth volume in the scanning session to correct for the head motions. No individual runs had more than 2 mm maximum movement in the x, y, z direction and 2° displacement in rotation for pitch, yaw, roll. The functional images were corrected for differences in slice timing to the middle slice. The data were then coregistered with the anatomical images and normalized to the standard T1 template volume (Montreal Neurological Institute, Montreal, Quebec, Canada). The images were then smoothed with an 8mm isotropic Gaussian kernel.

Statistical analyses at the first level and the second level were calculated on functional images acquired in the rhyming task using block design. Two contrasts were entered: activation of phonological processing (rhyming > orthography) (R-O) and activation of orthographic and phonological processing in rhyming task (rhyming > fixation) (R-F). A high-pass filter with cutoff period of 128 s was applied. BOLD signals were modeled using canonical HRF. Contrast images produced from the first level analysis were entered into random-effects analysis to obtain group results. All whole brain results are reported at a false discovery rate (FDR) correction p < 0.01 with a threshold more than 20voxels. The results from the group analyses were then used for choosing the regions of interest (ROIs) for effective connectivity analysis.

### Effective connectivity analysis

We used DCM [11] tool which is implemented in SPM8, to explore effective connectivity. DCM combines a neurobiological model of neural dynamics and a biophysical forward model that links neuronal activity to the measured signals [11, 59]. In DCM, three sets of parameters were estimated: the direct influence of stimuli on regional activity; the intrinsic or latent connections that describe couplings among regions and can be viewed as the “baseline” connectivity that is present in the system in the absence of external input; and the changes in the intrinsic connectivity between regions induced by experimental factors (modulatory effects) [11, 48]. We established a two-stage analysis: Subject-specific, first level DCMs were fully and reciprocally connected (12 connections), with modulatory (bilinear) effects of the rhyming conditions; the parameters from the first level DCM models were taken to a second level. In the second level analysis, BMS is used to test a set of alternative models and identify the most likely model. The BMS procedure takes into account both model accuracy and model complexity to determine the best model [13]. Bayesian estimation is used to make inferences about the parameters, which are expressed as the rate or speed of change (in Hz) of activity in one region that is associated with activity in another. We used BMS to select the best model and the BMA method to estimate the parameters of the winning model. To perform BMS, the fixed-effects analysis was used.

Four peak coordinates from the group analysis were used to guide the selection of ROIs for each subject: LIFG (-42, 42, 0), LMFG (-54, 12, 33), LIPL (-45, -39, 42) and LpMTG(-51, -48, 3). The time-course of each ROI for each subject was extracted in a sphere (radius: 6 mm) centered on the nearest local maxima (within the distance of 8 mm) from the coordinates mentioned above using the EIGENVARIATE function embedded in SPM. To ensure the consistency of the anatomical region through all the subjects, we applied Brodmann template of LIFG, LMFG, LIPL and LMTG as masks when the time-courses were extracted. LIPL’s responses were assigned as the driven input and were extracted from the R-F contrast. The responses of the other three regions were extracted from the R-O contrast. To include more significant active voxels in the sphere, we adjusted the uncorrected p to 0.05 for each subject. Two subjects were excluded, because there were no significant active voxel in the selected areas.

A total of 4096 (2^12^ = 4096) DCMs were specified for four regions each subject. For the DCM models, full and reciprocal connections were specified among the four ROIs. Input condition was modeled as exerting direct effect on the LIPL. The modulation effect was the rhyming task. During modeling, three types of parameters were calculated for each subject: 1) intrinsic connections between regions in the absence of modulating experimental effects; 2) the rhyming modulatory effect on intrinsic connections. 3) input to LIPL.

For the model comparison at the group level, sets of all DCMs for all subjects were submitted to a fixed effects Bayesian model comparison procedure [12]. The parameters of the winning model were computed by the BMA method and the connections were tested by one-sample T test across the subjects.

## Results

### Behavioral result

The reaction time (RT) was between 930.1 ms and 1886.1 ms (mean: 1365.2±274.7ms) in the rhyming trials and the RT was between 681.9 ms and 1443.0 ms (mean: 853.5±182.3ms) in the control trials for each subject. The accuracy percentages were above 75% (mean: 91.4%±10.1%) and above 92.5% (mean: 99%±1.9%) in the rhyming and the control trials for each subject. The mean RT in rhyming trials was longer than control trials in paired t-test (t = 8.9, p<0.01); however, the mean accuracy percentage in control trials was greater than that in the rhyming trials in paired t-test (t = 3.6, p<0.01).

### Imaging result

**Fig 2:**
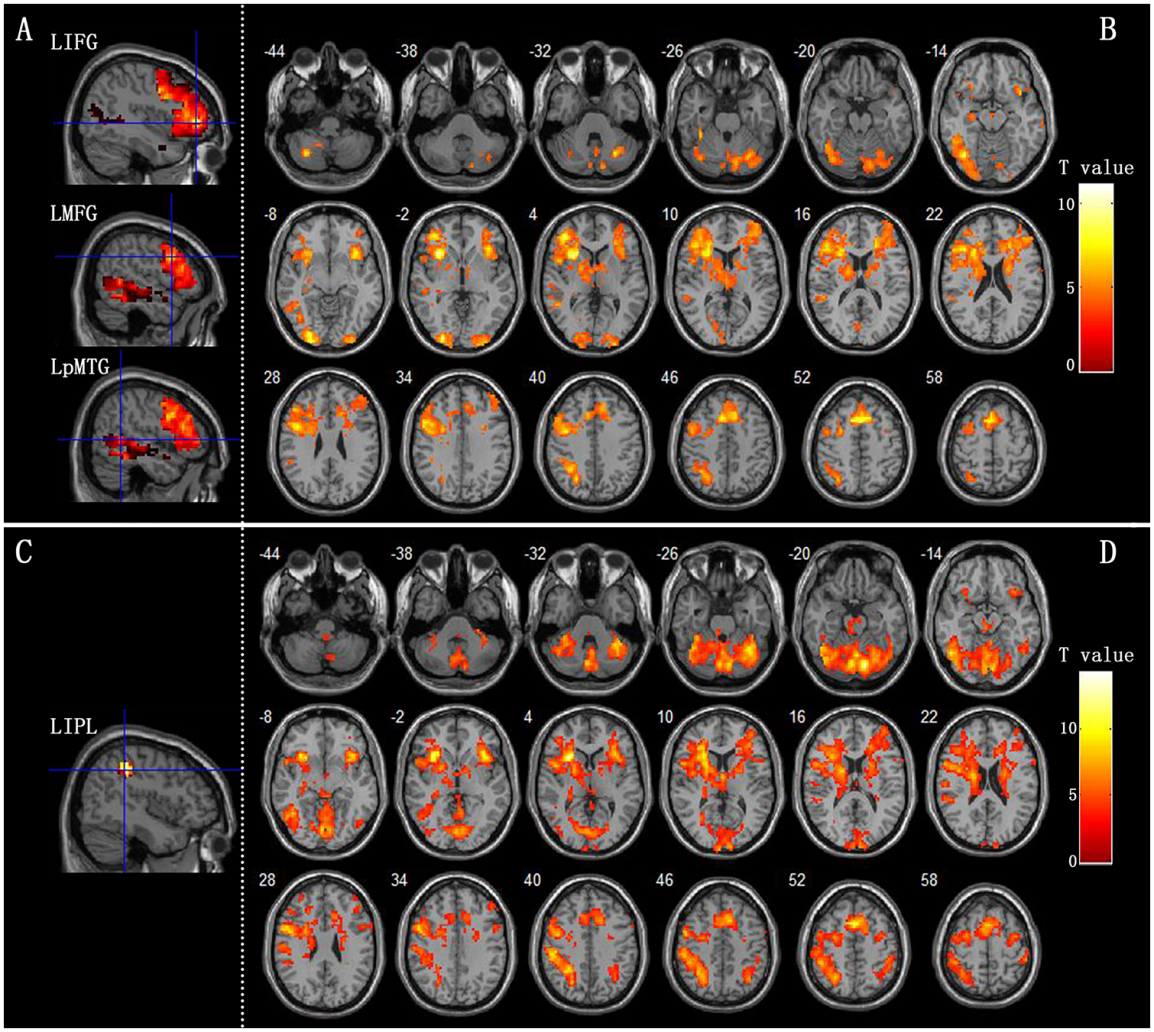
The activation maps of contrast R-O and contrast R-F. A: The peak points of LIFG, LMFG and LpMTG showed in the R-O result map with the mask of LIFG, LMFG and LMTG. B: The activation map of contrast R-O C: The peak points of LIPL showed in the R-F result map with the mask of LIPL.D: The activation map of contrast R-F

Fig 2 and Table 1 present the patterns of activations in the R-O and the R-F. The group maxima of the clusters (FDR p<0.01, extent threshold>20 voxel) were preserved. Subjects activated a neural network that was mostly lateralized to the left in the rhyming judgment task.

**Table 1:** The activation of contrast R-O and R-F.

Anatomical region	Hemisphere	Cluster peak (x, y, z)* (mm)	T score	Cluster size
***Contrast R-O*:**				
**Inferior frontal gyrus & middle frontal gyrus & insula**	L/R	−33 21 −3	11.08	4869
**Inferior occipital gyrus &inferior temporal gyrus & fusiform**	L	−27 −93 −6	10.90	759
**Cerebellum**	R	27 −66 −30	7.75	422
**Middle occipital gyrus**	R	30 −96 −6	7.21	191
**Cerebellum**	L	−33 −63 −45	7.21	22
**Inferior/Supperior Parietal Lobule**	L	−33 −51 39	7.20	349
**Middle Temporal Gyrus**	L	−51 −48 3	6.08	90
**Hippocampus**	L	−30 −15 −12	5.35	20
**Middle/Superior Temporal Gyrus**	L	−51 −30 0	4.92	30
***ContrastR-F*:**				
**Inferior/Middle/Superior Frontal Gyrus &Cerebellum&Inferior Parietal Lobule&Lingual Gyrus& insula &Precentral/Postcentral Gyrus&Fusiform &Middle Occipital Gyrus& Inferior/Middle/Superior Temporal Gyrus**	L/R	−30 18 3	14.34	10925
**Inferior Parietal Lobule**	R	33 −60 42	7.13	262
**Inferior/Middle Frontal Gyrus**	R	51 9 30	6.16	75

*The coordinates (x, y, z) correspond to MNI coordinates.

In the group activation patterns of phonological processing (R-O), subjects mainly activated neural circuits involving large clusters in the LMFG, LIFG, LpMTG, LpSTG, LIPL and inferior occipital lobe. The R-F produced a similar activation pattern to the R-O but more activation in left IPL, Cerebellum and left inferior/superior frontal gyrus. We used LMFG, LIFG, LpMTG/pSTG and LIPL to compute effective connectivity, because these four regions are highly correlated with language based on previous researches.

### Effective connectivity analysis results

**Fig 3:**
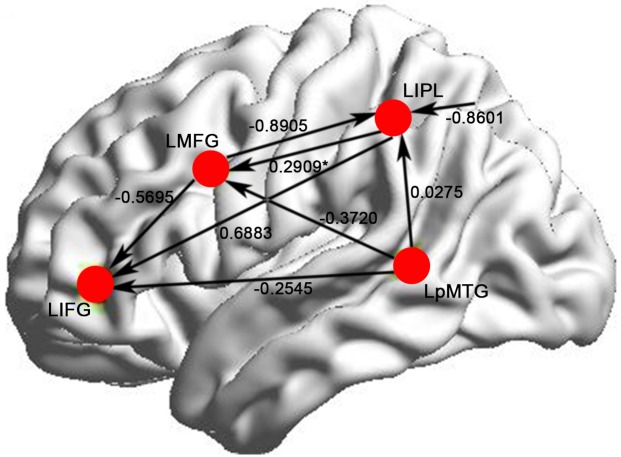
The winning model and the modulatory effects of the rhyming task. LIFG: left inferior frontal gyrus. LMFG: left middle frontal gyrus. LIPL: left inferior parietal lobule. LpMTG: left posterior middle temporal gyrus. The modulation marked by “*” is not significant.

Using DCM and BMS, the best model (model posterior probability = 1.0000) and the mean intrinsic connection of the model were computed and showed in Fig 3 and Table 2, respectively. Fig 3 also shows the modulatory effects of the rhyming task. There were modulatory effects of phonological load on the connections among these regions. In the winning model, LMFG had modulatory effects on LIPL (-0.8905) and LIFG (-0.5695) under rhyming judgment context; LIPL had modulatory effects on LIFG (0.6883) and LMFG (0.2909), but the modulatory effect from LIPL to LMFG is not significant (p < 0.05) across all the subjects; And LpMTG modulated LIFG (-0.2545), LMFG (-0.3720) and LIPL (0.0275) under rhyming judgment task.

**Table 2:** Mean intrinsic connection between the four ROIs.

To	From			
	LIFG	LIPL	LMFG	LpMTG
LIFG	**0.0822**	**0.1345**	0.0225	**0.0888**
LIPL	**0.1413**	**0.0840**	0.0121	**0.0981**
LMFG	**0.0799**	**0.0954**	**0.0481**	**0.0704**
LpMTG	**0.1145**	**0.1059**	0.0096	**0.0871**

Mean connections in bold are significant (p< 0.05).

## Discussion

Previous neuroimaging studies have established the connectivity analysis in alphabetic language, and showed that the areas involved in phonological processing were modulated by each other [50–52]. Bitan et al. found that the top-down control processes (IFG->lateral temporal cortex) in phonological task increase by development [50]. In this work, we explored the brain effective connectivity in Chinese for rhyming tasks. We firstly conducted a rhyming task and verified the activation pattern and then estimated the effective connectivity in the four important regions (LIFG, LMFG, LpMTG and LIPL) for Chinese rhyming tasks. Subjects mainly activated neural circuits involving large clusters in the LMFG, LIFG, LpMTG, left pSTG, LIPL and inferior occipital lobe. The more regions engaged in the phonological processing than those in orthographical one were consistent with the shorter RT and higher accuracy percentage. The activation pattern is similar with previous studies [7, 8, 16, 60].

The DCM and BMS methods were employed to estimate the effective connectivity and favor the best model. The full and reciprocal connections were specified to indentify the effective connectivity model for phonological processing without any prior information. BMS favored the best model from the 4096 models. Using DCM and BMS methods, we can get the dynamic directed causal influence among the specified regions [48]. We found that LMFG exerted inhibitory modulation on LIPL and LIFG; the phonological processing enhanced the connection from LIPL to LIFG and LMFG, which suggested the important roles of these connections for the increased phonological load; And LpMTG modulated LIFG and LMFG negatively, and LIPL positively under rhyming judgment task.

The first finding of our study was the significantly positive modulatory connection from LIPL to LIFG under rhyming task. The LIPL and LIFG play paramount roles in the phonological processing [18, 33]. A meta-analysis study proposed a fronto-parietal loop for phonological working memory [61] and more and more studies confirmed the proposal [8, 18]. A study on structural connectivity between left fronto-parietal brain regions showed that the left IPL and LIFG were connected by a dorsal pathway and the dorsal pathway provided a route for mapping from phonological memory in IPL to the inferior frontal articulatory network[62]. The positive modulatory connection from LIPL to LIFG supports the fronto-parietal loop for phonological working memory [62] and the activation of LIPL promotes the activation of LIFG. The phonological task would positively modulate the connection from LIPL to LIFG. For phonological processing, the interaction between LIPL and LIFG becomes more active. And the modulation on the connection from LIPL to LMFG varies across subjects.

The LMFG has been revealed as the special region in Chinese processing. In this work, we obtained causal connectivity from LMFG to LIFG and LIPL under rhyming tasks. LMFG was demonstrated to play a role of mapping the orthography to phonology of Chinese [45–47], and might be more engaged to integrate visual orthographic and phonological information when visual-orthographic load became heavier [63]. The negative modulations on the connections from LMFG to LIPL and LIFG elucidates that the dependence of LMFG on LIPL and LIFG decrease during the phonological decoding. As the positive modulatory connection from LIPL to LIFG supports the fronto-parietal loop for phonological working memory, it is reasonable that LMFG takes on the function of mapping orthography to phonology.

The phonological processing modulates the connection from LpMTG to LIPL positively, and from LpMTG to LIFG and LMFG negatively. The connectivity pattern of LpMTG demonstrated its important role for phonological processing. It was shown that LpMTG played a function of orthography and phonology mapping [45, 47]. Bitan et al. conducted the rhyming task experiment and found the middle portion of lateral temporal cortex was specified for the phonology processing [49]. The temporooccipital area may be tuned to the phonological properties of words and responsible for the feedback of phonology to orthography [8]. The connectivities from LpMTG to other regions support this point. The feedback information in LpMTG could reduce the connection to LMFG and LIFG and initiate the processing of LIPL.

In conclusion, the rhyming experiment and modulatory connectivity among the important brain regions in processing rhyming tasks were established in this study. It was found that LMFG exerted inhibitory modulation on LIPL and LIFG; the phonological processing enhanced the connection from LIPL to LIFG and LMFG, which suggested the important roles of these connections for the increased phonological load; And LpMTG modulated LIFG and LMFG negatively, and LIPL positively under rhyming judgment task.

## Limitation and Future Work

The change of BOLD signal relies heavily upon the resting blood volume fraction (V_0_) associated with regional vasculature. As shown in the study of Hu et al., the employment of an assumed V_0_ in fMRI data assimilation is only suitable for fMRI signal reconstruction and activation detection grounded on this signal, and not suitable for estimation of unobserved states and effective connectivity study [64]. They introduced a physically realistic V_0_ for more accurate estimation [64] and a quantitative method for evaluating the activation state in fMRI images [65]. In this work, we don’t get enough information to calculate V_0_ and its impact on the effective connectivity. The default value of V_0_ was used in the analysis. For future work, the impact of V_0_ on the phonological processing network should be investigated.

## Supporting Information

S1 File The minimal data set. (RAR)

S1 Table The model parameters of all subjects. (DOCX)
