# Supplementary material for: The Brain Effective Connectivity of Chinese during Rhyming Task
Source: PLoS One. 2016 Sep 1;11(9):e0162158. doi: 10.1371/journal.pone.0162158 (PMC5008726; doi:10.1371/journal.pone.0162158)
Supplement: S1 Table — (DOCX) [file pone.0162158.s002.docx]

S1 Table. The model parameters of all subjects

| Subject ID | IPL->IFG | MFG->IFG | MTG->IPL | MFG->IPL | MTG->IPL | IPL->MFG | MTG->MFG |
| --- | --- | --- | --- | --- | --- | --- | --- |
| 1 | -0.0018 | -0.0048 | 0.0219 | 0.0079 | -0.0234 | -0.0142 | 0.0006 |
| 2 | 0.0054 | 0.0043 | -0.0078 | -0.0058 | 0.0097 | 0.0157 | -0.0119 |
| 3 | 1.3232 | 0.5513 | -0.8333 | -1.3455 | -0.5735 | -1.0258 | -0.6508 |
| 4 | 1.1034 | 0.5095 | -1.0640 | -1.9077 | -0.3799 | 0.7621 | -0.6784 |
| 5 | 1.3338 | 0.6013 | -1.2086 | -2.0035 | -0.5777 | 0.4451 | -0.7263 |
| 6 | 1.4810 | 0.6232 | -1.1990 | -1.9596 | -0.6513 | 1.2301 | -0.8709 |
| 7 | 1.1364 | 0.5039 | -1.0232 | -1.6523 | -0.4499 | -0.5156 | -0.6200 |
| 8 | -0.0049 | -0.0038 | 0.0172 | 0.0145 | -0.0183 | -0.0073 | -0.0069 |
| 9 | 1.5956 | 0.5509 | -1.4109 | -1.9395 | -0.4031 | 1.4734 | -0.7392 |
| 10 | 1.4403 | 0.6414 | -1.0378 | -1.2609 | -0.4079 | -1.2292 | -0.7582 |
| 11 | 0.0017 | 0.0095 | -0.0078 | -0.0047 | -0.0101 | -0.0070 | -0.0093 |
| 12 | 0.0073 | -0.0035 | 0.0175 | -0.0031 | 0.0071 | 0.0164 | -0.0058 |
| 13 | 0.7969 | 0.4168 | -0.7424 | -1.1824 | -0.2472 | -0.5760 | -0.5681 |
| 14 | 1.5090 | 0.5745 | -1.2064 | -1.8823 | -0.5916 | -0.0747 | -0.6593 |
| 15 | -0.0144 | -0.0053 | 0.0075 | -0.0132 | 0.0009 | -0.0254 | -0.0050 |
| 16 | -0.0057 | -0.0134 | 0.0084 | -0.0034 | -0.0101 | -0.0123 | -0.0076 |
| 17 | -0.0065 | -0.0112 | -0.0130 | 0.0026 | 0.0006 | 0.0128 | -0.0064 |
